# Address before the Suffolk District Medical Society, (Mass.)

**Published:** 1850-08

**Authors:** John Jeffries

**Affiliations:** President of the Society


*Address before the Suffolk District Medical Society*, (*Mass.*) by John Jeffries, M. D., President of the Society.

This is a very able and elegant production. The subject is, the "relative position of the medical profession with the public." The causes affecting the estimation in which the profession is held by the public are admirably sketched, and the influences that have tended to the improvement of medical science and art are well considered.

